# Doxorubicin induces cardiotoxicity through upregulation of death receptors mediated apoptosis in cardiomyocytes

**DOI:** 10.1038/srep44735

**Published:** 2017-03-16

**Authors:** Liqun Zhao, Baolin Zhang

**Affiliations:** 1 Office of Biotechnology Products, Center for Drug Evaluation and Research, Food and Drug Administration, Silver Spring, MD 20993, USA

## Abstract

Doxorubicin is a highly effective anticancer agent but causes cardiotoxicity in many patients. The mechanisms of doxorubicin-induced cardiotoxicity remain incompletely understood. Here we investigated doxorubicin-induced cytotoxicity in human induced pluripotent stem cells-derived cardiomyocytes (iPS-CMs). We found that doxorubicin and related anthracycline agents (e.g., daunorubicin, idarubicin, and epirubicin) significantly upregulated the expression of death receptors (DRs) (TNFR1, Fas, DR4 and DR5) in iPS-derived cardiomyocytes at both protein and mRNA levels. The resulting iPS-CMs cells underwent spontaneous apoptosis which was further enhanced by physiologically relevant death ligands including TNF-related apoptosis inducing ligand (TRAIL). Furthermore, TRAIL potentiated doxorubicin-induced decrease in beating rate and amplitude of iPS-derived cardiomyocytes. These data demonstrate that the induction of death receptors in cardiomyocytes is likely a critical mechanism by which doxorubicin causes cardiotoxicity.

Doxorubicin is widely prescribed for the treatment of solid tumours (e.g., breast, ovary and gastrointestinal) and haematologic malignancies (e.g., lymphoma and leukemia) in both adults and children. While an effective anti-tumor agent, doxorubicin causes cumulative and dose-dependent cardiotoxicity, ranging from occult changes in myocardial structure and function to severe cardiomyopathy and congestive heart failure that may result in cardiac transplantation or death^1^,^2^,^3^,^4^,^5^,^6^. The exact causal mechanisms of doxorubicin-induced cardiotoxicity remain elusive, making it difficult to predict or prevent its severe adverse event in individual patients^7^,^8^,^9^.

Doxorubicin exerts its anti-tumoral activity primarily by intercalation into DNA and inhibiting topoisomerase II (TOP2) in fast-proliferating cancer cells, leading to cancer cell death^10^. Similarly, doxorubicin cardiotoxicity features apoptosis or other forms of cell death in cardiomyocytes, resulting in loss of functional myocytes and irreversible heart injury. However, the cardiotoxicity of doxorubicin appears separable from its therapeutic mechanism because cardiomyocytes are generally not replicative, and Top2α, the primary target of doxorubicin, is not expressed in quiescent cells and undetectable in heart tissues^11^,^12^. Generation of reactive oxygen species (ROS) is a classical mechanism by which doxorubicin injuries the myocardium^3^,^13^. However, there is also evidence suggesting that ROS is unlikely to be the primary mechanism of doxorubicin-induced cardiotoxicity^14^. Other proposed mechanisms include impaired mitochondrial function, disruption of Ca^2+^ homeostasis, and altered gene and protein expression that triggers cell death^4^,^7^.

Death receptors (DRs), including TNF receptor 1 (TNFR1), Fas, DR4 and DR5, are crucial mediators of apoptosis under physiological conditions. Individual DRs can be activated by their cognate ligands TNFα, Fas ligand (FasL), and TNF-related apoptosis-inducing ligand (TRAIL) that are produced by various types of cells (e.g., T cells, Natural Killer cells) and are normally present in blood and tissue microenvironment. Besides the paired partners of TNFα/TNFR1 and FasL/Fas, TRAIL selectively binds DR4 and DR5 expressed on cell surface. Ligation of DRs induces the assembly of a death inducing signaling complex (DISC) that triggers activation of a caspase cascade, cleavage of cellular proteins, and ultimately apoptosis of target cells^15^.

Doxorubicin was reported to induce the expression of DR4 and DR5 in many cancer types, thereby enhancing TRAIL induced apoptosis^16^,^17^,^18^,^19^,^20^,^21^,^22^. Moreover, doxorubicin upregulates Fas expression and enhances Fas-mediated apoptosis in several cancers^23^,^24^. Notably, DR-mediated apoptosis machinery is conserved in human cardiomyocytes^8^,^9^,^25^. Both Fas and FasL were increased in rat cardiomyocytes following doxorubicin treatment and associated with cardiac dysfunction in rat^26^,^27^,^28^. However, animal models may not accurately recapitulate doxorubicin-induced cardiotoxicity due to inter-species differences in both the drug metabolism and cardiac structure and function. In particular, significant differences between mouse and human cardiac system in terms of electrophysiology and contractile features limit the extrapolation of findings from studies in murine systems to humans^29^,^30^. Recently, human pluripotent stem cell-derived cardiomyocytes (hiPS-CMs) have emerged as a powerful tool to model cardiac toxicity in highly physiologically relevant human cells^31^,^32^,^33^. In this study, we investigated drug-induced cardiotoxicity using iPS-CMs assay system. We made novel findings to implicate DR-mediated apoptosis in doxorubicin-induced cardiotoxicity.

## Results

### Doxorubicin induces apoptotic cell death in iPS-derived cardiomyocytes

**Figure 1:**
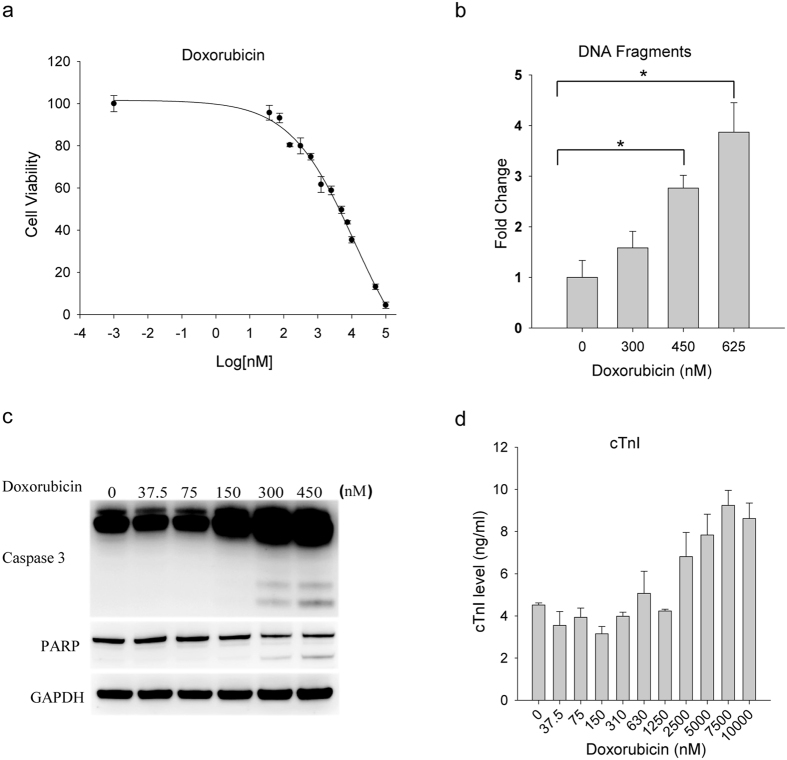
Doxorubicin induces apoptotic cell death in iPS-derived cardiomyocytes. iPS-CMs were plated onto 96 plates at 30,000 cells per well and treated with an increasing doses of doxorubicin for 48 h. The resulting cells were analyzed for cell viability (**a**), DNA fragmentation (**b**), caspase activation (**c**), and cTnI release (**d**). Error bars represent means ± SD for triplicate experiments. *p < 0.01.

In clinical settings, doxorubicin can induce cell death of cardiomyocytes in some patients within hours of intravenous administration^34^. We investigated doxorubicin-induced cytotoxicity in iPS-derived cardiomyocytes. Cells were grown onto fibronectin coated plates and exposed to doxorubicin at increasing doses. The resulting cells were analyzed using orthogonal assays for cell viability, apoptosis, and the release of cardiac factors. As expected, doxorubicin treatment resulted in a dose-dependent decrease in cell viability with an IC50 of 3.5 μM (Fig. 1a). An increase in apoptotic cell population was detected by the positive immunostaining of active caspase-3 (data not shown), an increase in DNA fragmentation (Fig. 1b), and caspase activation as indicated by the cleavage of caspase 3 and its substrate PARP (poly ADP ribose polymerase)^35^ (Fig. 1c). Further, there was a simultaneous release of cardiac troponin I (cTnI) (Fig. 1d) – a biochemical marker that is clinically used for the diagnosis of myocardial injury^36^,^37^. Notably, doxorubicin’s cytotoxicity in the iPS-CMs was evident at doses as low as ~0.5 μM, which is even lower than the peak concentration range of the drug observed in plasma of patients after intravenous administration^38^. Overall, these data are in agreement with previous reports from cellular and human biopsy studies demonstrating doxorubicin-induced cardiotoxicity^33^. The data support the use of the iPS-derived cardiomyocytes as a human cellular model to assess the potential cardiotoxicity of anticancer agents.

### Doxorubicin potently induces the expression of death receptors in iPS-derived cardiomyocytes

**Figure 2:**
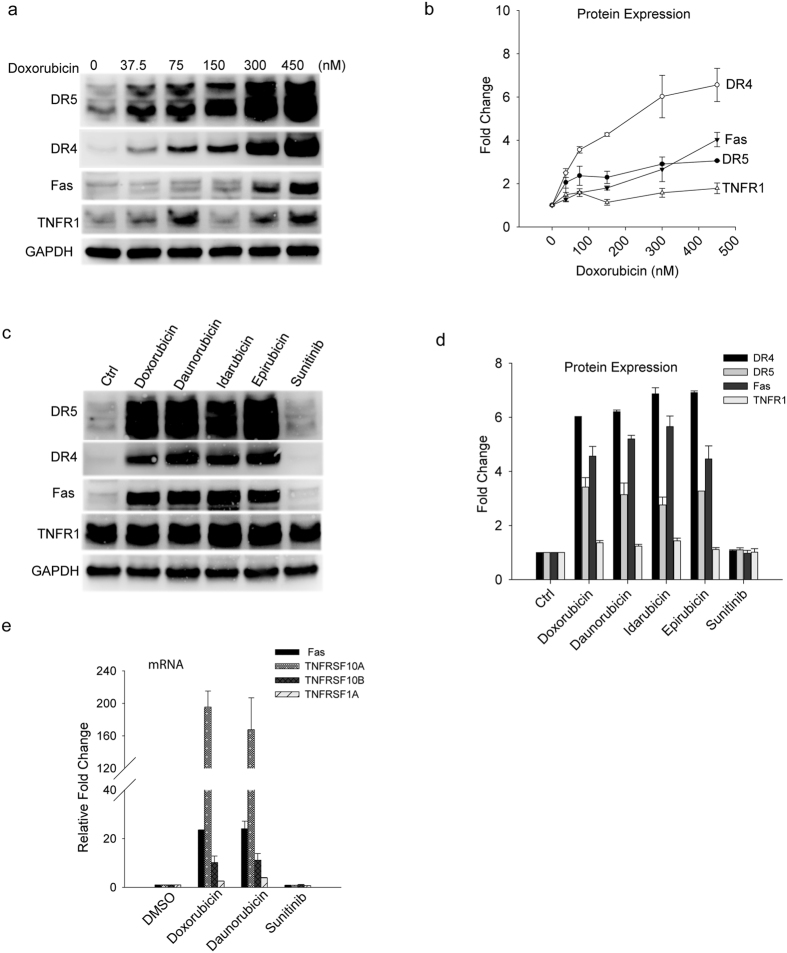
Anthracyclines upregulate the expression of death receptors in iPS-CMs. (**a**) iPS-CMs cells were exposed to the increasing doses of doxorubicin for 48 h. Whole cell lysates were analyzed by western blotting using antibodies specific to DR5, DR4, Fas, and TNFR1, respectively. (**b**) Densitometry analysis of the bands in (**a**). Shown are the fold increases in protein expression levels of TNFR1, Fas, DR4 and DR5 relative to those in the untreated cells. (**c**) Immunoblots for the DR proteins from iPS-CMs treated with individual anthracyclines (doxorubicin, daunorubicin, idarubicin or epirubicin) at 0.26 μg/ml or sunitinib at 0.14 μg/ml for 48 h. (**d**) Densitometry analysis of the bands in (**c**) yielding the relative fold increases in individual DR protein expression. (**e**) mRNA expressions of DRs in iPS-CMs treated with the indicated drugs (doxorubicin, daunorubicin or sunitinib) relative to the untreated cells. Error bars represent mean ± SD for triplicate experiments.

To gain insight into the molecular mechanisms involved in doxorubicin induced cardiotoxicity, we determined the expression of death receptors in iPS-derived cardiomyocytes. Cells were treated with doxorubicin at increasing doses for 48 hours and the resulting cells were lysed and analyzed by immunoblotting. Strikingly, doxorubicin induced a dose-dependent increase in the protein expression levels of all four DRs tested including TNF receptor 1 (TNFR1), Fas, DR4, and DR5 (Fig. 2a). At 450 nM, all four DRs were significantly upregulated with the most profound effect being seen for DR4 (~7 times increase) (Fig. 2b). A similar observation was made for structurally related compounds within the anthracycline family, including daunorubicin, idarubicin, and epirubicin (Fig. 2c,d), which are known to cause cardiotoxicity^2^. By sharp contrast, sunitinib (a tyrosine kinase inhibitor) showed little or no effect on any of the DRs tested.

At the transcriptional level, the mRNA expression of all four DRs displayed at least a 2-fold increase in iPS-derived cardiomyocytes treated with doxorubicin or daunorubicin (Fig. 2e). The mostly pronounced effect was seen for DR4 (*TNFRSF10A*) whose mRNA expression was elevated approximately 170 times compared to its baseline level in the control cells (Fig. 2e). The expression of several pro-apoptotic genes such as caspase 3, caspase 8, FADD was also elevated, albeit to a lesser extent (data not shown). Again, none of the DRs was affected in their mRNA levels after sunitinib treatment (Fig. 2e). These data demonstrate that doxorubicin, as well as related anthracycline family compounds, potently induce the expression of DRs at both mRNA and protein levels in iPS-derived cardiomyocytes. In cancer cells, overexpression of DRs was shown to cause receptor clustering on cell membrane thereby triggering activation of a caspase cascade and subsequent apoptosis^39^. A similar mode of action may occur in iPS-CMs upon doxorubicin treatment, leading to spontaneous apoptosis of the cardiomyocytes (Fig. 1). The induction of DRs in iPS-CMs reveals a novel mechanism by which doxorubicin may induce cardiotoxicity.

### TRAIL enhanced Doxorubicin-induced cytotoxicity in iPS-derived cardiomyocytes

**Figure 3:**
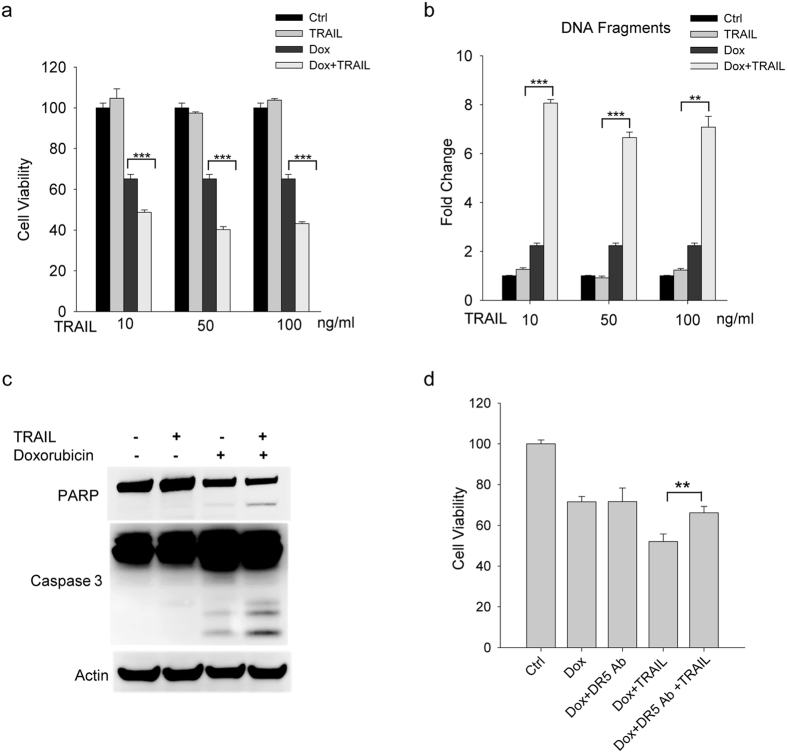
TRAIL enhances doxorubicin-induced cytotoxicity in iPS-derived cardiomyocytes. (**a**–**c**) iPS-CMs were pre-treated with 450 nM of doxorubicin for 24 h and were then supplemented with a fresh medium containing 450 nM of doxorubicin and TRAIL at the indicated concentrations (**a**,**b**). The cells were incubated for another 24 h and analyzed for cell viability (**a**) and DNA fragmentation (**b**). **(c)** Immunoblots showing the cleavage of PARP and caspase-3 in iPS-CMs treated with doxorubicin (450 nM) and TRAIL (50 ng/ml) alone or in combination as described above. (**d**) iPS-CMs were pretreated with doxorubicin (450 nM) alone or in combination with a DR5 neutralizing antibody (10 μg/ml) for 24 h, followed by incubation with TRAIL (50 ng/ml) for another 24 h. The results show an inhibitory effect of DR5 neutralizing antibody on TRAIL-induced cytotoxicity. Error bars represent mean ± SD for triplicate experiments. **p < 0.001; ***p < 0.0001.

**Figure 4:**
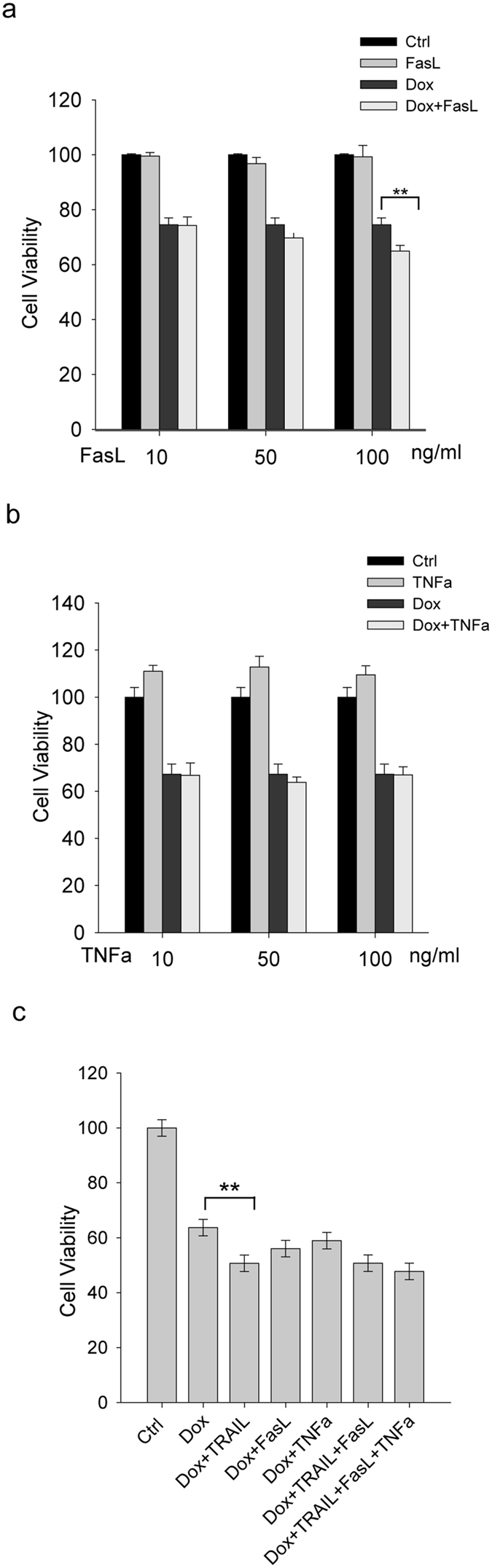
FasL and TNFα displayed minor effects on doxorubicin-induced cytotoxicity in iPS-derived cardiomyocytes. After treatment with doxorubicin (450 nM) for 24 h, iPS-CMs were incubated in a fresh medium containing doxorubicin (450 nM) and FasL (**a**) or TNFα (**b**) at the indicated doses, or in (**c**) with a fixed dose of each death ligand (50 ng/mL) for another 24 h. Error bars represent mean ± SD for triplicate experiments. **p < 0.001.

Under physiological conditions, individual DRs respond to their cognate ligands (e.g. TRAIL, TNFα or FasL) that are largely produced by immune cells and present in circulation^40^. There is also evidence that doxorubicin induced the release of FasL from rat cardiomyocytes, which may provide feedback signaling to kill target cells through the upregulated DRs^27^,^28^. To mimic such a physiological relevant condition, cells were treated with doxorubicin for 24 hours followed by incubation with individual death ligands (TRAIL, TNFα or FasL) at varying doses (10, 50 and 100 ng/ml) for another 24 hours (Fig. 3 and 4). When combined with doxorubicin, TRAIL stimulated a further reduction in cell viability of iPS-CMs that was accompanied by a robust increase in apoptosis index and caspase activity (Fig. 3b,c). Notably, this cytotoxicity effect was effectively blocked by pretreating the cells with a DR5 neutralizing antibody (Fig. 3d). This data supports the notion that DR5 upregulation may be a critical mechanism in doxorubicin induced cardiotoxicity. By contrast, FasL and TNFα showed little or no effect on cell viability when combined with doxorubicin (Fig. 4a,b) while both ligands remained active to induce apoptosis in cancer cell lines (data not shown). This was further confirmed when the iPS-CMs were treated with a combination of the death ligands (Fig. 4c). The inclusion of FasL or TNFα or both FasL and TNFα failed to introduce any further change in cell viability compared to TRAIL treatment.

**Figure 5:**
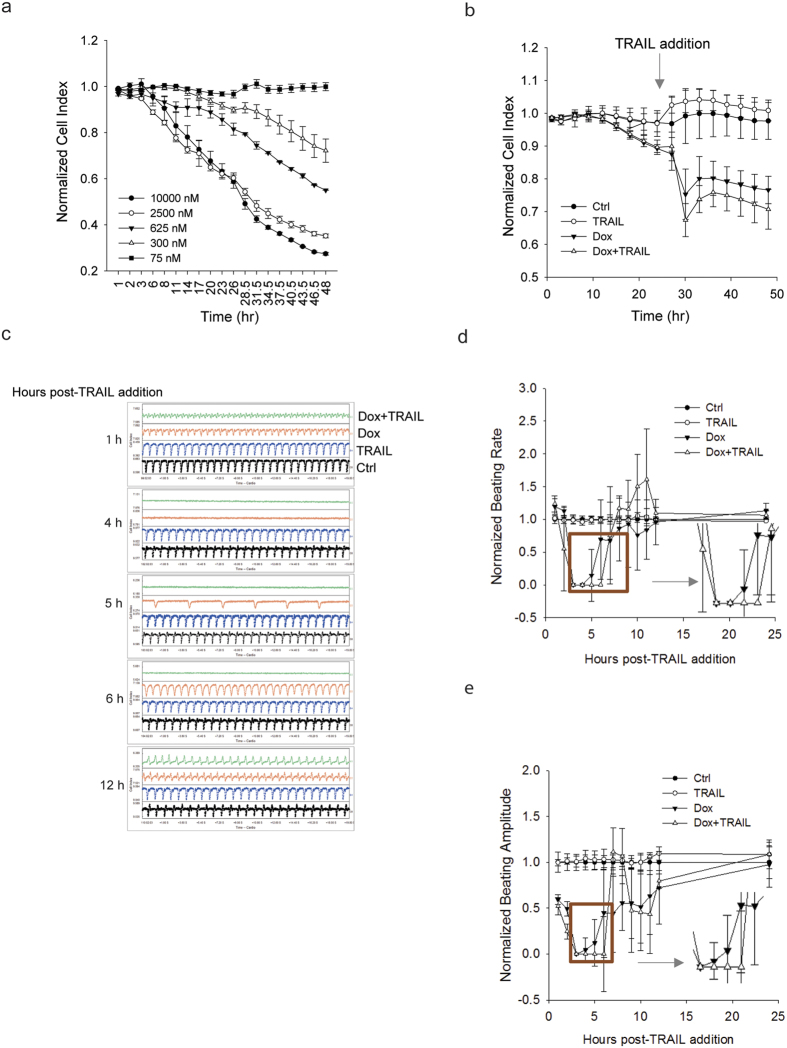
TRAIL augments doxorubicin-induced alterations in electrophysiological properties of iPS-derived cardiomyocytes. (**a**) iPS-CMs cells were cultured in fibronectin-coated microplates and treated with doxorubicin (450 nM) at the increasing doses for 48 h. Cells were monitored for real-time cell index changes using xCELLIgence RTCA Cardio system. (**b**–**e**) iPS-CMs were pretreated with doxorubicin (450 nM) for 24 h followed by addition of TRAIL (10 ng/mL), as indicated by arrow in **b**. The resulting cells were monitored for cell index (**b**), beating patterns (**c**), beating rate (**d**) and beating amplitude (**e**). *Note:* the time courses shown in d and e are post TRAIL treatment. Error bars represent mean ± SD for triplicate experiments.

Clinical use of doxorubicin has been associated with various arrhythmias by affecting the electrophysiological characteristics of cardiomyocytes^41^. To gain insight into electrophysiological effects of the drug and TNF cytokines, we assessed the beating properties of iPS-CMs treated with doxorubicin in the absence or presence of TRAIL through impedance measurement using xCELLigence Cardio platform. Consistent with the data in Fig. 1, doxorubicin treatment induced a time- and dose-dependent decrease in cell viability as shown by the normalized cell index (Fig. 5a). The addition of TRAIL resulted in a further decrease in cell viability (Fig. 5b). The resulting iPS-CMs displayed distinct patterns in beating rate and beating amplitude (Fig. 5c). The beating signals of iPS-CMs were completely arrested at 4 h and thereafter appeared at 5 h and returned to normal beating pattern at 8 h post TRAIL treatment. Intriguingly, there was a delay in the recovery of beating patterns in the iPS-CMs after co-treatment with doxorubicin and TRAIL. In those cells, no beating signals were detected until 8 h post-treatment as shown in the time course of normalized beating rate and amplitude (Fig. 5d,e). Taken together, these data suggest that TRAIL might be directly involved in the execution of doxorubicin induced cardiotoxicity under physiologically relevant conditions.

## Discussion

**Figure 6:**
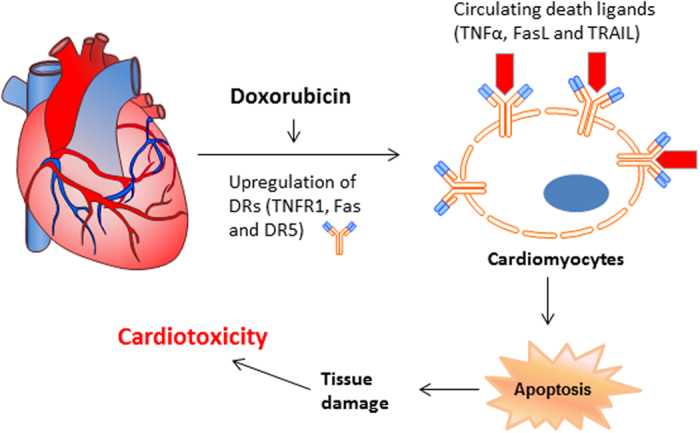
A working model by which doxorubicin induces cardiotoxicity through upregulation of death receptors mediated apoptosis in cardiomyocytes. Doxorubicin and related anthracyclines appear to be potent inducers of the expression of death receptors (TNFR1, Fas, DR4 and DR5) in cardiomyocytes. The upregulated DRs may undergo clustering or engage their cognate ligands, thereby triggering a caspase cascade and ultimate apoptosis in cardiomyocytes. The elevated serum levels of specific TNF cytokines (e.g., TRAIL), which could occur under certain disease and treatment conditions, may be predictive of the risk of cardiotoxicity in individual patients prior to the administration of doxorubicin or anthracycline agents.

A body of evidence establishes iPS-derived cardiomyocytes as an *in vitro* assay system to model drug-induced cardiotoxicity^42^,^43^. This human cellular model recapitulates many of the known cardinal features of cardiotoxicity observed in humans thereby allowing a physiologically accurate dissection of the adverse drug action^33^. Using iPS-CMs assays, we identified a novel mechanism by which doxorubicin elicits toxicity in cardiomyocytes. This involves an upregulation of death receptors (TNFR1, Fas, DR4 and DR5) and subsequent apoptosis in iPS-derived cardiomyocytes. Similar effects were also found for other anthracyclines, including daunorubicin, idarubicin, and epirubicin. In contrast, little or no effect on DR expression was detected in iPS-CMs when treated with sunitinib (Fig. 2) or several other anticancer agents including Herceptin, 5-FU, and Taxol (data not shown). Notably, doxorubicin-induced cytotoxicity was further potentiated in the presence of TNF cytokines such as TNF-related apoptosis inducing ligand (TRAIL). Anthracyclines appear to potently induce the expression of death receptors in cardiomyocytes, resulting in spontaneous or ligand-dependent apoptosis in target cells. These data suggest that the elevated serum levels of specific TNF cytokines could be predictive of the risk of doxorubicin-induced cardiotoxicity in individual patients (Fig. 6). Additional studies are warranted to evaluate the causal relationship between the baseline serum levels of specific TNF cytokines and the adverse cardiac events in patients receiving doxorubicin treatment. These data also support the utility of the iPS-CMs cellular system for high throughput screening of novel cardioprotective agents to mitigate the cardiotoxicity of doxorubicin and other anthracyclines.

Several biochemical pathways are associated with doxorubicin-induced cardiotoxicity; including increased production of reactive oxygen species (ROS), disruption of intracellular calcium homeostasis, inhibition of topoisomerase II-β (TOP2β) and anti-apoptotic proteins (e.g., Bcl-X_L_), or induction of pro-apoptotic proteins (e.g., Bax)^44^,^45^. These cellular events are thought to trigger intrinsic apoptosis in cardiomyocytes. Under physiological conditions, TNF-related cytokines are known to play an important role in maintaining tissue homeostasis. These cytokines can engage their corresponding death receptors (TNFR1, Fas, DR4, and DR5) expressed on cell membrane, leading to activation of a caspase cascade and ultimate cell death – a process known as extrinsic apoptosis. In some cancer cells, doxorubicin was shown to induce the expression of DR4 and DR5 that was thought to at least partly contribute to the drug’s anticancer activity^20^,^21^,^22^. Notably, the extrinsic apoptosis machinery is conserved in cardiomyocytes across animal species. Doxorubicin treatment increased Fas/FasL expression in rat cardiomyocytes^27^,^46^,^47^ as well as human iPS-CMs^48^. TNFα/TNFR1 was also shown to be elevated in rat cardiomyocytes following doxorubicin treatment, but its relevance to cardiac injury remains elusive. One report suggested that the elevated TNFR1 expression was positively associated with doxorubicin-induced apoptosis in rat cardiomyocytes^49^. This was in contrast to an observation made in TNFR1 knockout mice suggesting a role for TNF/TNFR1 signaling in protecting cardiomyocytes from doxorubicin’s toxicity^50^. Here we show a significant increase in all four DRs tested, including TNFR1, Fas, DR4 and DR5, at both mRNA and protein levels in the iPS-derived cardiomyocytes upon doxorubicin treatment (Fig. 2). The upregulated DRs may undergo clustering on the cell surface, leading to spontaneous apoptosis (Fig. 1). At present, the precise mechanism by which doxorubicin upregulates DR transcripts is not clear but it may involve activation of NF-κB pathway. As a shared transcription factor for all four DRs, NF-κB was shown to be activated in multiple cancer types in response to doxorubicin treatment^15^,^51^,^52^. Nonetheless, the ability of doxorubicin to upregulate DRs in cardiomyocytes provides critical insight into the molecular mechanisms of the adverse drug reaction.

The above findings could help identify biomarkers to predict the risk of cardiotoxicity in individual patient prior to the administration of doxorubicin or other anthracyclines. Currently, multiple strategies are clinically used for prevention and early detection of anthracycline-induced cardiomyopathy^53^. These include the measurement of serum cardiac troponins (cTnT and cTnI) that are closely associated with the severity of myocardial damage in patients treated with doxorubicin or other chemotherapy^54^,^55^. The elevated TnT levels were shown to be predictive of subsequent left ventricular dilation and left ventricular wall thinning identified by echocardiography^56^. However, it is recognized that a broad range of conditions are associated with the raised cTnT and cTnI values which causes diagnostic confusion and clinical dilemmas in patient management. Moreover, the serum cTnT and cTnI levels are only raised after cardiac damage or dysfunction had occurred. There is an unmet need for biomarkers that can predict potential cardiotoxicity before the onset of adverse cardiac events^57^. Our data show that the TNF-related cytokines, in particular TRAIL, could augment doxorubicin-induced cardiotoxicity by engaging the upregulated death receptors on the cardiomyocytes (Fig. 3 and 4). Compared to TRAIL, FasL and TNFα only showed little or no effect on doxorubicin-induced cytotoxicity in the iPS-derived cardiomyocytes. However, these observations were made under the specific *in vitro* conditions with recombinant death ligand proteins. Given that both Fas and TNFR1 are upregulated in iPS-CMs by doxorubicin, the *in vitro* data does not rule out the possibility that FasL/Fas and TNF/TNFR1 pathways contribute to doxorubicin induced cardiotoxicity *in vivo*. In light of the presence of TNF cytokines in circulation, whose serum levels can be very different among patients based on the status of disease and the history of treatment, we hypothesize that the elevated serum levels of TNF cytokines could help identify patient populations at a higher risk to doxorubicin associated cardiotoxicity. Investigations are underway in our laboratory to evaluate the causal relationship between the baseline serum levels of specific TNF cytokines and the adverse cardiac events in cancer patients receiving doxorubicin treatment.

## Materials and Methods

### Cell culture of iPS-derived cardiomyocytes

Human induced pluripotent stem cell-derived cardiomyocytes (iPS-CMs), named Cor.4U, were obtained from Axiogenesis (Germany) and were cultured onto fibronectin-coated wells of 96-well plates following vendor’s protocols. The plates were prepared by coating each well with 50 μl of 10 μg/ml fibronectin (FN) solution (F1114, Sigma-Aldrich), and incubated at 37 °C for 3 hr. Meanwhile, frozen vials of Cor.4U cells were thawed and diluted with pre-warmed Cor.4U Culture Medium at 300,000 cells per ml. After removing the excess coating solution, each well was supplemented with 100 μl of the cell suspension (30,000 cells per well). The plates containing Cor.4U cells were kept at room temperature for 30 min and then maintained at 37 °C in a humidified incubator supplied with 5% CO2. Culture medium was refreshed daily without disturbing cell attachment. Under these culture conditions, Cor.4U cells normally will produce robust and regular beating signal patterns on Day 3.

### Reagents and Antibodies

Trastuzumab (Herceptin, Roche) and 5-FU (Fluorouracil, Fresenius Kabi) were purchased from the Division of Veterinary Resources pharmacy, Office of Research Services, National Institute of Health (Bethesda, MD). Doxorubicin hydrochloride (Catalog #D1515), epirubicin hydrochloride (E9406), idarubicin hydrochloride (I1656) and sunitinib malate (PZ0012) were purchased from Sigma-Aldrich (St. Louis, MO). Daunorubicin hydrochloride (1467) was from Tocris Bioscience (Minneapolis, MN). Monoclonal antibodies specific to Fas (sc-8009) and TNF-R1 (sc-8436) were from Santa Cruz Biotechnology (Dallas, TX). Western antibodies against human DR5 (8074), DR4 (D9S1R), PARP (9542), and caspase 3 (9662) were from Cell Signaling Technology (Danvers, MA). DR5 neutralizing antibody (CDM234) was purchased from Cell Sciences (Newburyport, MA). GAPDH (NB300–328) antibody was from Novus Biologicals (Littleton, CO). Recombinant human death ligand proteins TRAIL/TNFSF10 (rhTRAIL) (375-TEC) and TNFα (654205) were from R&D Systems (Minneapolis, MN), Fas ligand (ALX-522–020) was from Enzo Life Sciences (Farmingdale, NY).

### Cytotoxicity assays

Cor.4U cells were seeded onto fibronectin-coated 96-well plates at 30,000 cells per well and incubated at 37 °C for 3 days when robust beating signals were reached. On Day 4, cells were treated with doxorubicin or other drugs (*see* details in Figure legends). Cell viability was determined using CellTiter 96 AQ_ueous_ One Solution Cell Proliferation Assay kit from Promega (Madison, WI). The levels of lactate dehydrogenase^25^, a cytosolic enzyme that is released into culture medium upon plasma membrane damage, were determined using a LDH Cytotoxicity Assay Kit from ThermoFisher Scientific (Rockville, MD). The release of troponin I (cTnI), a commonly used protein biomarker of cardiac damage, was measured using Troponin I (Human) ELISA Kit from Abnova (Taipei, Taiwan) as previously described^43^.

### Apoptosis assay

Apoptosis index was assessed using Cell Death Detection ELISA^PLUS^ from Roche Life Science (Indianapolis, IN) to measure the levels of histone-associated-DNA fragments in cytosol upon apoptosis induction. Briefly, cells were lysed by adding 250 μl lysis buffer and centrifuged at 200 g for 10 min, 20 μl of supernatant was transferred to the streptavidin-coated microplate. Meanwhile, 80 μl immunoreagent containing anti-histone-biotin and anti-DNA-POD (peroxidase) was added to each well and incubate for 2 hours at room temperature. The plate was washed three times with incubation buffer and 100 μl of enzyme substrate solution was pipetted to each well to incubate for 10–20 min until color was developed. 100 μl stop solution was added and absorbance at 490 nm was read. The OD values in treated samples were compared with those values obtained from untreated control cells, and expressed as apoptosis fold changes.

### Western blotting

Whole cell lysates were prepared using RIPA buffer (Sigma-Aldrich). After centrifugation at 14000 rpm for 15 min, supernatants were collected and analyzed for protein concentration using BCA Protein Assay Kit (ThermoFisher Scientific). Equal amounts of total proteins were resolved onto SDS-PAGE using 4–12% NuPAGE Bis-Tris gel (Life Technologies), transferred to PVDF membranes, incubated with specific antibodies, and visualized by ECL reagents. The immunoblots were subjected to densitometry analysis using the LAS-4000 Luminescent Image Analyzer (Fujifilm).

### Functional data recording and analysis

Cor.4U cardiomyocytes were seeded at 30,000 cells per well in a fibronectin-coated E-Plate Cardio 96 (ACEA Biosciences) and cultured as described above. When Cor.4U cells generated robust and regular beating signals, which usually occurs on Day 3, drug treatment was initiated on the following day (Day 4). Four hours prior to treatment, culture medium was refreshed with 90 μl of pre-warmed Cor.4U CM maintenance medium followed by adding 90 μl of 2-fold drug solution at different concentrations. The resulting cells were analyzed for cell index, beating rate and amplitude using the xCELLigence RTCA Cardio system (ACEA Biosciences). The baseline signal in the untreated Cor.4U cells was acquired through 20-second recording at 5 min intervals for 30 min. The cellular response to drug treatments was recorded every 5 min (20-second recording) in the first hour, and afterwards, every 60 min. After the data acquisition, the RTCA Cardio software is used to calculate the parameters such as normalized beating rate and amplitude with statistical analysis.

### Quantative PCR array

Quantitative real-time PCR (qRT-PCR) was performed using RT^2^ Profiler PCR Array Human Apoptosis containing 84 apoptosis-related genes (Cat#330231, Qiagen). Basically, total RNAs were extracted using QIAGEN RNeasy Kit (74104). Five housekeeping genes were included on the array (B2M, HPRT1, RPLP0, GAPDH, and ACTB) to normalize the RNA amounts. Relative fold changes in gene expression were calculated based on the threshold cycle (Ct) value using the formula 2^(−ΔΔCt)^.

### Statistical analysis

All cell-based assays were performed in triplicates and results are presented as mean ± Standard Deviation^58^. Statistical significance was analyzed by Student’s *t* test using Sigmaplot software (Systat Software Inc., San Jose, CA). p-value < 0.01 indicates a significant difference between two data values.

## Additional Information

**How to cite this article**: Zhao, L. and Zhang, B. Doxorubicin induces cardiotoxicity through upregulation of death receptors mediated apoptosis in cardiomyocytes. *Sci. Rep.* **7**, 44735; doi: 10.1038/srep44735 (2017).

**Publisher's note:** Springer Nature remains neutral with regard to jurisdictional claims in published maps and institutional affiliations.
